# Supplementary material for: The interactome of intact mitochondria by cross-linking mass spectrometry provides evidence for coexisting respiratory supercomplexes
Source: Mol Cell Proteomics. 2017 Dec 8;17(2):216–32. doi: 10.1074/mcp.RA117.000470 (PMC5795388; doi:10.1074/mcp.RA117.000470)
Supplement: Supplemental Data [file supp_RA117.000470_133922_0_supp_23978_fzffwf.zip › spectra_annotation/mito_DR_spectra_annotation/210-1-10-1-7-1.pdf]

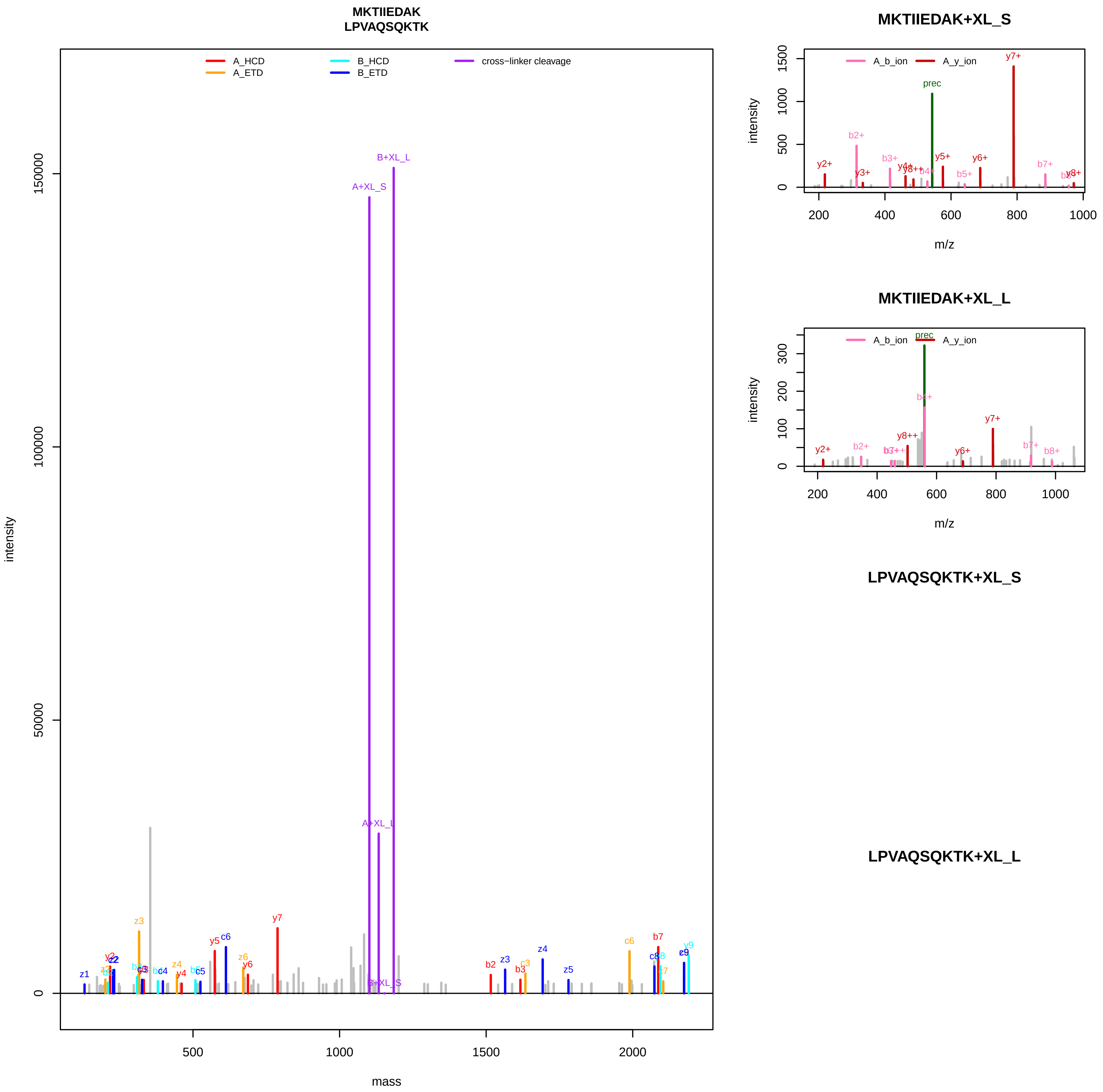
MKTIIEDAK
LPVAQSQKTK
A_HCD
A_ETD
B_HCD
B_ETD
cross−linker cleavage
B+XL_L
A+XL_S
A+XL_L
B+XL_S
intensity
mass
0
50000
100000
150000
500
1000
1500
2000
z1
y2
c2
z3
y7
y5
c6
z6
y6
z4
y4
c5
c4
c3
b2
b3
c3
z5
c6
b7
c8
z7
y9
z9
MKTIIEDAK+XL_S
A_b_ion
A_y_ion
prec
y7+
b2+
b3+
y2+
y3+
y4+
y8++
b4+
y5+
y6+
b5+
b7+
y8+
m/z
MKTIIEDAK+XL_L
b8+
LPVAQSQKTK+XL_S
LPVAQSQKTK+XL_L
